# Long-Term Survival in a Case of Primary Leiomyosarcoma of the Inferior Vena Cava in Which PET-CT Follow-up Was Useful

**DOI:** 10.3400/avd.cr.20-00056

**Published:** 2020-09-25

**Authors:** Masahiro Aiba, Ikutaro Kigawa

**Affiliations:** 1 Department of Cardiovascular Surgery, Totsuka Kyoritsu Second Hospital

**Keywords:** leiomyosarcoma, inferior vena cava, PET-CT

## Abstract

Leiomyosarcoma of the inferior vena cava (IVC) is a rare malignancy, but has been found more frequently with recent advances in diagnostic imaging. Local recurrence and metastases are frequent with this pathology, and prognosis is poor. We report a case of a patient with leiomyosarcoma of the IVC surviving for >10 years after the first resection despite local recurrence and two metastatic recurrences to the pancreas and liver, with successful excisions following early detection on positron emission tomography-computed tomography.

## Introduction

Leiomyosarcoma of the inferior vena cava (IVC) is a rare malignancy of the venous system originating from smooth muscle cells of the middle layer of the IVC. Although a number of cases have been reported with recent advances in diagnostic imaging, local recurrences and/or distant metastases are often seen, and the disease shows poor prognosis. We report a case of a patient with long-term survival after the first resection of leiomyosarcoma of the IVC, with successful excisions of subsequent local recurrence and two metastatic recurrences after early detection on positron emission tomography (PET)-computed tomography (CT).

## Case Report

**Figure 1:**
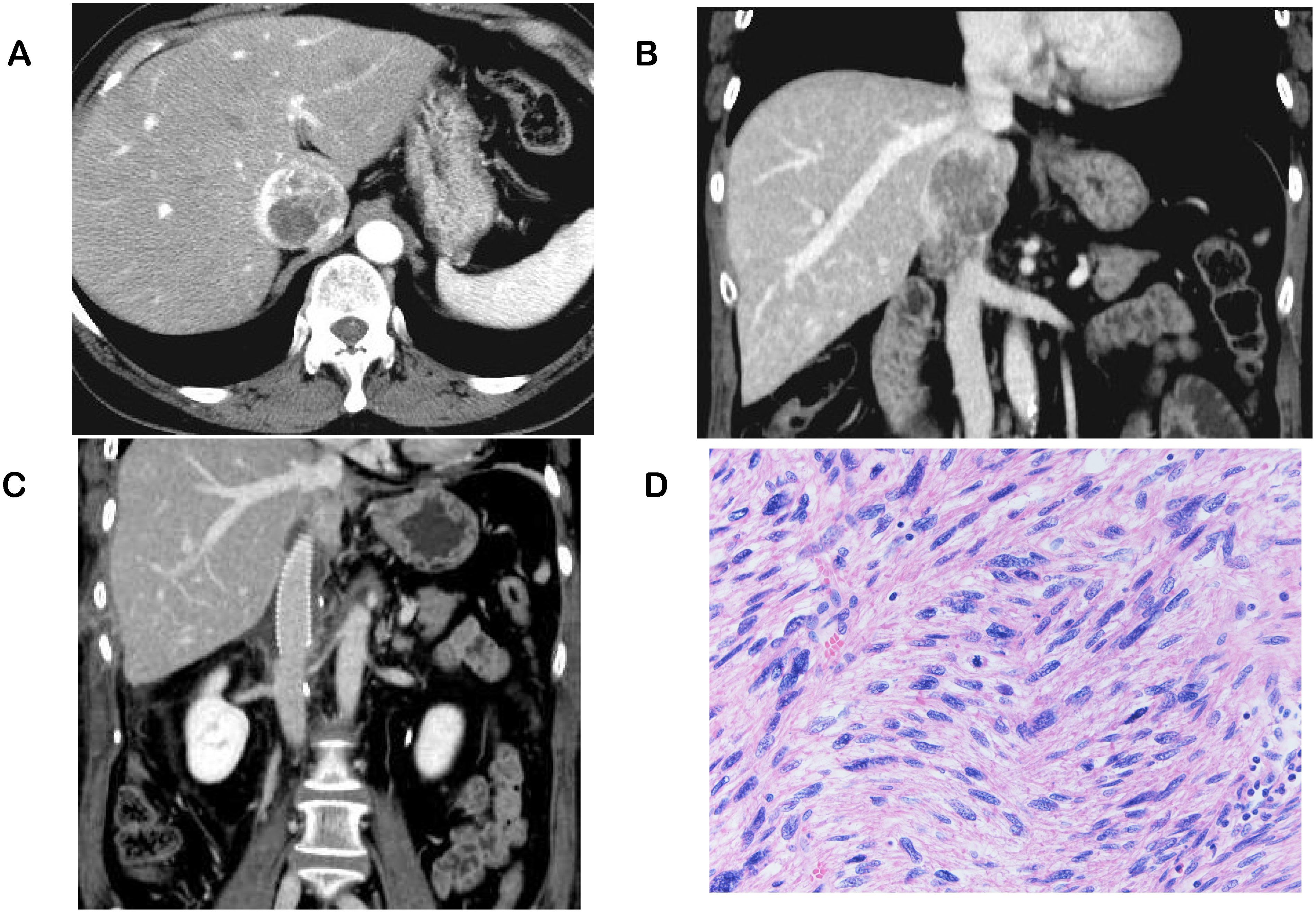
Fig. 1 (**A**) Preoperative dynamic enhanced computed tomography (CT) (axial). The inferior vena cava (IVC) at the level of the liver is dilated, showing a tumor shadow in the lumen. (**B**) Preoperative dynamic enhanced CT (coronal). The tumor is present in the proximal IVC from the renal vein junction to the hepatic vein junction. (**C**) Postoperative contrast-enhanced CT shows the replaced graft. (**D**) Microscopic findings of the excised tumor. The lesion comprises fascicles of spindle cells with blunt-ended nuclei. The pathological diagnosis is leiomyosarcoma originating from the IVC.

A 58-year-old woman at the time of the first operation was referred after identification of a tumor embolus inside the hepatic vein on abdominal ultrasonography during a medical examination. The tumor was suspected to be leiomyosarcoma of the IVC localized to the middle segment on dynamic CT, and surgery was performed (**Figs. 1A** and **1B**). The segment of the IVC from the renal vein junction to the diaphragm was approached using an epigastric region L-shape incision and de-rolling of the liver. First, the IVC and bilateral renal veins were clamped on the distal side, but no decrease in blood pressure was observed. Then, the IVC on the proximal side of the hepatic vein junction was clamped after performing hilar clamping with the Pringle technique. The tumor had originated in the venous wall of the middle segment of the IVC, reaching the porta hepatis and growing into the lumen. The tumor with the IVC was widely excised with adequate margins. The proximal clamp was then transferred to the IVC in the lower liver to release the hilar clamp, and the resected vessel was replaced by an artificial graft (expanded polytetrafluoroethylene (ePTFE); 16 mm with ring) by simple clamping (**Fig. 1C**). She was discharged 20 days postoperatively without any major complications.

Microscopically, the lesion comprised fascicles of spindle-shaped cells with blunt-ended nuclei and was diagnosed pathologically as leiomyosarcoma originating from the IVC (**Fig. 1D**). Excisional margins were negative for tumor cells.

**Figure 2:**
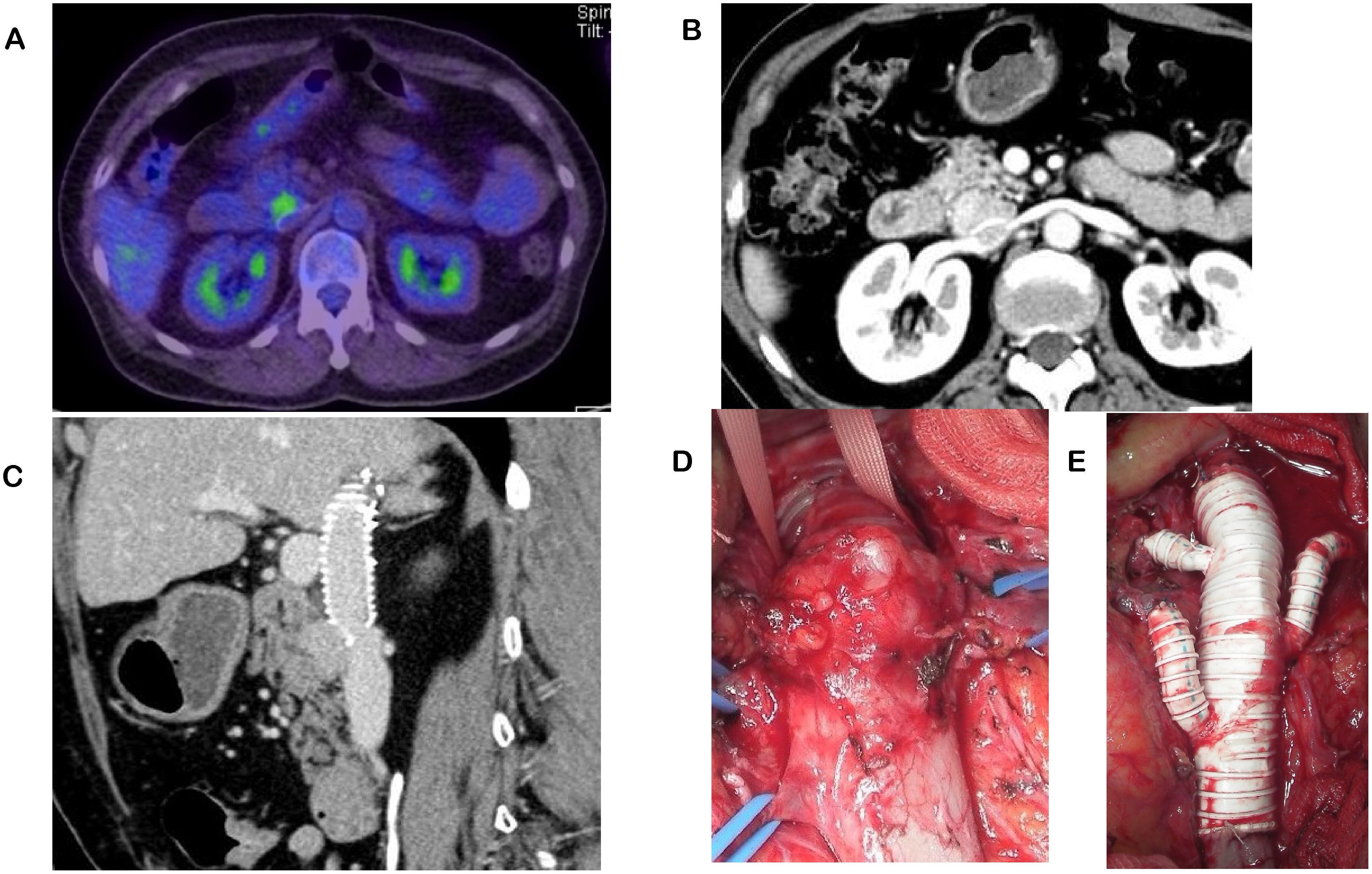
Fig. 2 (**A**) Positron emission tomography (PET)-computed tomography (CT) 2 years after the first operation shows an intense accumulation of fluorodeoxyglucose (FDG) at the anastomosis on the distal side of the graft. (**B**) Contrast-enhanced CT reveals an extramural mass approximately 2 cm in diameter growing on the lower side of the head of the pancreas. (**C**) The tumor is present on the front of the site of anastomosis between the graft and inferior vena cava (IVC). (**D**) The intraoperative image shows the tumor has grown to outside the IVC. The capsule is strong, and the pancreas and neighboring organs show no macroscopic infiltration. (**E**) The IVC is replaced, and the renal veins (double right renal veins and single left vein) are reconstructed using a branched graft.

No adjuvant therapy such as chemotherapy or radiation was performed. After discharge, the patient underwent annual follow-up PET-CT and magnetic resonance imaging (MRI). Two years after the first operation, PET-CT showed an intense accumulation of fluorodeoxyglucose (FDG) at the anastomosis on the distal side of the graft, and tumor recurrence was suspected (**Fig. 2A**).

Contrast-enhanced CT revealed an extramural mass approximately 2 cm in diameter growing on the lower side of the pancreas head at the distal anastomosis of the IVC and artificial graft. The border of the tumor and pancreas was relatively clear, with no apparent invasion to the surrounding organs (**Figs. 2B** and **2C**).

Local recurrence of leiomyosarcoma of the IVC was diagnosed, and a reoperation was performed. Midline incisions were made from the previous skin incision to the center of the inferior abdomen, and the IVC segment above the renal vein junction was exposed by de-rolling the descending duodenum to the left side. No tumor was present in the IVC lumen, but tumor growth to outside the IVC was evident, extending to the front of the anastomosis of the ePTFE graft and IVC (**Fig. 2D**). The tumor capsule remained strong, and the pancreas and neighboring organs did not show macroscopic infiltration. Detachment was thus easy. Under intermittent clamping of the IVC and renal veins, part of the initial graft and IVC was widely excised with the tumor, and the IVC was replaced and the renal veins (double right renal veins and single left vein) were reconstructed using a branched ePTFE graft (16 mm and 6 mm with ring) that had been made during the operation (**Fig. 2E**). Postoperative contrast-enhanced CT revealed occlusion of the reconstructed left renal vein, but collateral circulation to the gonadal vein was established and no problems were evident. The patient was again discharged with no other major complications 16 days postoperatively.

**Figure 3:**
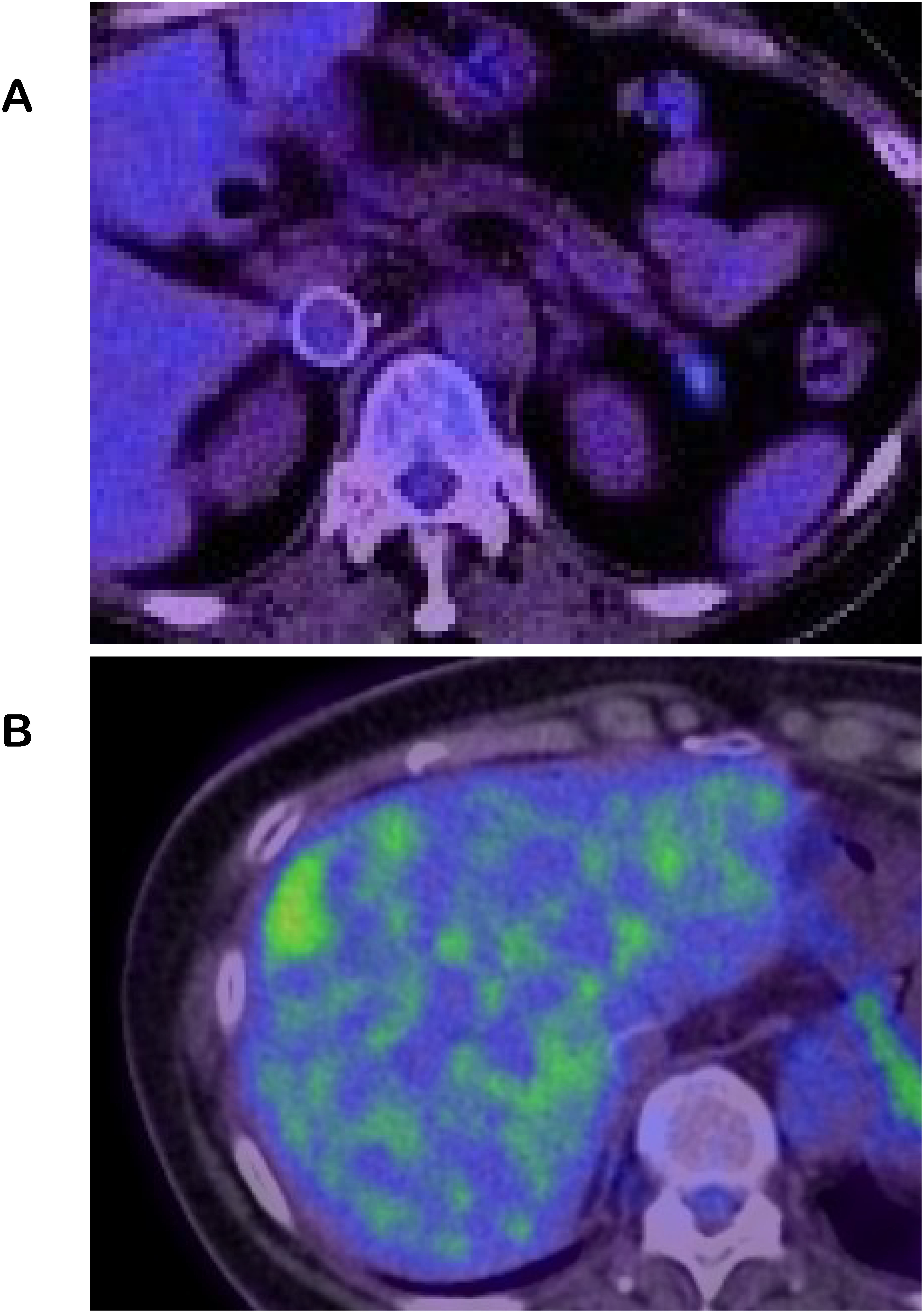
Fig. 3 (**A**) Positron emission tomography (PET)-computed tomography (CT) at 9 months after the second operation shows an abnormal accumulation of fluorodeoxyglucose in the pancreas tail. (**B**) PET-CT at 6 years and 4 months after resection of the tail of the pancreas shows abnormal accumulation in the liver (S8).

Nevertheless, PET-CT performed 9 months after this second operation revealed abnormal accumulation in the tail of the pancreas. Resection in this region was performed at another hospital (**Fig. 3A**). The specimen was pathologically diagnosed as leiomyosarcoma.

After performing follow-up PET-CT every year thereafter, PET-CT at 6 years and 4 months after resection of the pancreas tail showed abnormal accumulation in the S8 area of the liver (**Fig. 3B**). A partial hepatectomy was performed at the previous hospital. Pathologically, the tumor was again diagnosed as leiomyosarcoma.

The patient has been examined by PET-CT every year since the last operation, with no local recurrence recognized as of 12 years after the first resection of leiomyosarcoma of the IVC. As of 10 years after local recurrence, the patient remains alive and well.

## Discussion

Leiomyosarcoma of the IVC is a rare malignancy originating from smooth muscle cells of the media with intra- or extraluminal growth. This entity was first reported by Perl in 1871,^1)^ and several cases have been described with recent advances in diagnostic imaging. In Japan, 68 cases were reported from 1987 to 2020, based on a search of the Japan Medical Abstract Society (excluding proceedings). However, local recurrences and metastases are frequent, and prognosis is poor with this pathology. Wachtel et al. analyzed and reported 377 cases of initial resection of a primary leiomyosarcoma of the IVC.^2)^ They described 1- and 5-year disease-free survival rates of 57% and 6%, respectively, with most patients experiencing recurrence, and 1- and 5-year overall survival (OS) rates of 92% and 55%, respectively. Long-term survival is possible, but survival for 15 years is achieved by only a significant minority. A number of distant metastases have been reported, but postoperative distant metastases involved several organs such as the lungs, thighs, shoulder muscles, liver, bone, subcutaneous nodules, peritoneum, breasts, external iliac veins, small bowel, abdominal wall, chest wall, and inguinal lymph nodes.^3)^

For the treatment of leiomyosarcoma of the IVC, the importance of radical resection of the primary tumor has been widely proposed.^4,5)^ However, although resection with positive margins has been reported to affect declines in OS,^2)^ local recurrence has been reported even with negative margins.^6)^ Furthermore, treatment of distant metastases does not show any clear tendency due to the extent of metastases; the tumor may have grown at the time of discovery, making radical surgery impossible, and good results have not been obtained in several cases, even with radio- or chemotherapy. On the contrary, some patients with long-term survival despite distant metastases have undergone aggressive resection.^4)^

For long-term survival, detecting local recurrences and distant metastases soon after primary resection is important, along with performance of aggressive treatment.^7)^ We also believe that the early detection of local recurrences and distant metastases can reduce the invasiveness of resection.

No tumor markers are currently available to accurately identify soft-tissue tumors, including leiomyosarcoma, and diagnostic imaging alone is the only option for early detection and clues to recurrences and metastases. Several reports have described Doppler ultrasonography and contrast-enhanced CT and MRI for the postoperative follow-up of leiomyosarcoma of the IVC. However, curative surgery may already be impossible when such imaging modalities raise the suspicion of recurrence.^7)^

PET-CT shows high accumulation in leiomyosarcoma of the IVC, and its diagnostic effectiveness has been reported.^8)^ Distinguishing between intravascular tumor and thrombus is difficult on MR angiography and contrast-enhanced CT. However, PET-CT can discriminate tumor from thrombus due to differences in metabolism; hence, tumor recurrence can be detected at an earlier stage. In addition, PET-CT is useful for staging sarcomas with a high probability of metastasis, offers superior sensitivity and specificity in detecting local recurrence compared with conventional imaging, and is highly useful for diagnosing distant metastases.^9)^

In this case, the resection margin was negative at the time of the first operation, but local recurrence and two distant metastases were then identified. PET-CT for postoperative follow-up was able to detect these lesions early, and complete resection was possible without any major invasion. Twelve years after the initial operation and 10 years after local recurrence, no further local recurrence of the IVC has been observed. Although distant metastases remain possible, follow-up is being actively conducted.

## Conclusion

If possible, PET-CT every year after surgery for leiomyosarcoma of the IVC allows the early detection of local recurrences and distant metastases. In addition, we believe that early detection can reduce the degree of surgical invasiveness, allowing radical resection and raising the chance of long-term survival.

## References

[1] Perl L, Virchow R. Ein Fall von Sarkom der Vena cava inferior. Archiv f pathol Anat 1871; 53: 378-83.

[2] Wachtel H, Gupta M, Bartlett EK, et al. Outcomes after resection of leiomyosarcomas of the inferior vena cava: a pooled data analysis of 377 cases. Surg Oncol 2015; 24: 21-7. 25433957 10.1016/j.suronc.2014.10.007

[3] Jeong S, Han Y, Cho Y, et al. Clinical outcomes of surgical resection for leiomyosarcoma of the inferior vena cava. Ann Vasc Surg 2019; 61: 377-83. 31394210 10.1016/j.avsg.2019.05.053

[4] Alkhalili E, Greenbaum A, Langsfeld M, et al. Leiomyosarcoma of the inferior vena cava: a case series and review of the literature. Ann Vasc Surg 2016; 33: 245-51. 26802297 10.1016/j.avsg.2015.10.016

[5] Mastoraki A, Leotsakos G, Mastoraki S, et al. Challenging diagnostic and therapeutic modalities for leiomyosarcoma of inferior vena cava. Int J Surg 2015; 13: 92-5. 25489949 10.1016/j.ijsu.2014.11.051

[6] Ayers B, Toth SA, Mix DS, et al. Surgical management of a recurrent leiomyosarcoma of the inferior vena cava requiring aortobifemoral bypass. J Vasc Surg 2017; 65: 93S.

[7] Ganeshalingam S, Rajeswaran G, Jones RL, et al. Leiomyosarcoma of the inferior vena cava: diagnostic features on cross-sectional imaging. Clin Radiol 2011; 66: 50-6. 21147299 10.1016/j.crad.2010.08.004

[8] Singh N, Shivdasani D, Karangutkar S. Rare case of primary inferior vena cava leiomyosarcoma on F-18 fluorodeoxyglucose positron emission tomography-computed tomography scan: differential from nontumor thrombus in a background of procoagulant state. Indian J Nucl Med 2014; 29: 246-8. 25400364 10.4103/0972-3919.142629 PMC4228588

[9] Lim HJ, Johnny Ong C-A, Tan JW-S, et al. Utility of positron emission tomography/computed tomography (PET/CT) imaging in the evaluation of sarcomas: a systemic review. Crit Rev Oncol Hematol 2019; 143: 1-13. 31449981 10.1016/j.critrevonc.2019.07.002

